# Thoracic Fluid Content as an Indicator of High Intravenous Diuretic Requirements in Hospitalized Patients with Decompensated Heart Failure †

**DOI:** 10.3390/jcm13185625

**Published:** 2024-09-22

**Authors:** Agata Galas, Paweł Krzesiński, Małgorzata Banak, Grzegorz Gielerak

**Affiliations:** Department of Cardiology and Internal Diseases, Military Institute of Medicine, National Research Institute, 04-141 Warsaw, Poland; agalas@wim.mil.pl (A.G.); mbanak@wim.mil.pl (M.B.); ggielerak@wim.mil.pl (G.G.)

**Keywords:** heart failure, hypervolemia, diuretics, diuretic resistance, hospitalization

## Abstract

**Background:** The main cause of hospitalization in patients with heart failure is hypervolemia. Therefore, the primary treatment strategy involves diuretic therapy using intravenous loop diuretics to achieve decongestion and euvolemia. Some patients with acutely decompensated heart failure (ADHF) do not respond well to diuretic treatment, which may be due to diuretic resistance (DR). Such cases require high doses of diuretic medications and combination therapy with diuretics of different mechanisms of action. Although certain predisposing factors for diuretic resistance have been identified (such as hypotension, type 2 diabetes, impaired renal function, and hyponatremia), further research is needed to identify other pathophysiological markers of DR. **Objective:** This study aims to identify admission markers that can predict a high requirement for intravenous diuretics in hospitalized patients with decompensated heart failure. **Methods:** This study included 102 adult patients hospitalized for ADHF. At admission, patients underwent clinical assessment, laboratory parameter evaluation (including the N-terminal prohormone of brain natriuretic peptide [NT-proBNP] levels), and hemodynamic assessment using impedance cardiography (ICG). Hemodynamic profiles were based on the use of parameters such as heart rate (HR), blood pressure (BP), and thoracic fluid content (TFC) as markers of volume status. The analysis included 97 patients with documented doses of intravenous diuretic use. Patients were stratified into two groups based on median diuretic consumption (equivalent to 540 mg of intravenous furosemide): the high-loop diuretic utilization (LDU) group (*n* = 49) and the low-LDU group (*n* = 48). **Results:** Compared to low-LDU patients, high-LDU patients had greater thoracic fluid content at admission, both quantitatively (37.4 ± 8.1 vs. 34.1 ± 6.9 kOhm-1; *p* = 0.024) and qualitatively (TFC ≥ 35 kOhm-1: 59.2% vs. 33.3%; *p* = 0.011). Anemia was more common in the high-LDU group (67.4% vs. 43.8%; *p* = 0.019), as was elevated NT-proBNP (≥median of 3952 pg/mL: 60.4% vs. 37.5%; *p* = 0.024). High LDU was associated with a significantly longer hospitalization duration (12.9 ± 6.4 vs. 7.0 ± 2.6 days; *p* < 0.001). Logistic regression analysis identified anemia, elevated NT-proBNP, and high TFC as predictors of high LDU (HR: 2.65, 2.54, and 2.90, respectively). In a multifactorial model, only high TFC remained an independent predictor (HR: 2.60, 95% CI 1.04–6.49; *p* = 0.038). **Conclusions:** TFC was the sole independent admission marker of a high requirement for intravenous diuretics in patients hospitalized for decompensated heart failure. An objective assessment of volume status by impedance cardiography may support intensive personalized decongestion therapy.

## 1. Introduction

Chronic heart failure (CHF) is a common progressive syndrome characterized by recurrent episodes of deterioration, often caused by hypervolemia [1,2,3]. Acute decompensated HF (ADHF) results in over one million hospitalizations annually and significantly contributes to morbidity and mortality [4,5,6]. Approximately 80–90% of ADHF patients have symptoms of fluid overload and require intensive treatment with loop diuretics to achieve decongestion [7,8]. The utilization of diuretic therapy in ADHF is difficult and should be tailored to each patient’s needs [7]. According to the REALITY-AHF trial, the prompt initiation of diuretic treatment is associated with lower in-hospital mortality [9]. Patients with de novo acute heart failure typically require lower doses compared to those already receiving furosemide for CHF. To assess early diuretic responses, the current guidelines recommend assessing spot urine sodium (UNa+) concentrations 2 h after the first diuretic dose [1,2,7]. Greater sodium excretion is associated with reduced mortality [10].

Before the era of natriuresis-guided therapy, diuretic resistance (DR) was defined as an inadequate response and ineffective decongestion despite maximum-dose diuretic therapy [11]. Ultimately, approximately 20% of patients may require escalated decongestion therapy [8,12]. Only a few risk factors for DR have been identified: type 2 diabetes, chronic kidney disease, atherosclerotic disease, hypotension, hyponatremia, and pneumonia [13]. It is imperative to anticipate the potential for DR at the onset of treatment, enabling the implementation of a more aggressive therapeutic approach from the outset. Therefore, we aimed to verify whether any of the other admission markers could be useful for the prediction of high intravenous diuretic requirements in patients hospitalized for ADHF.

## 2. Materials and Methods

This was a secondary analysis of the prospective observational study, which enrolled 102 adult patients of both sexes hospitalized for ADHF (defined according to the European Society of Cardiology guidelines [1,2]) and who required intravenous diuretic therapy at the Department of Cardiology and Internal Diseases of the Military Institute of Medicine between November 2014 and March 2017. The group consisted of both decompensated CHF and de novo HF patients. The exclusion criteria have been described in our previous papers [14,15]. Briefly, patients with unstable angina, a history of acute coronary syndrome and/or coronary artery bypass grafting surgery within the last 12 weeks, non-cardiogenic shock (i.e., sepsis or bleeding with hypotension requiring catecholamines), severe pulmonary hypertension or other severe lung conditions, pulmonary embolism, poorly controlled hypertension, acute and/or decompensated non-cardiovascular disease, valvular disease or other acquired heart defects requiring surgical intervention, hemoglobin < 10.0 g/dL, end-stage chronic kidney disease, and neoplastic disease were not eligible.

The study protocol was approved by the local bioethics committee (Bioethics Committee of the Military Medical Institute in Warsaw, Warsaw, Poland, approval no. 14/WIM/2012; 16 May 2012), and all study participants provided their written informed consent. This study was registered at ClinicalTrials.gov (NCT 02355769). The patients were treated according to the current guidelines.

The patients underwent detailed clinical, laboratory, and hemodynamic assessments upon admission. To assess the patients’ hemodynamic profiles, impedance cardiography (ICG, NiccomoTM device [Medis, Ilmenau, Germany]) was used. All ICG measurements were performed within 24 h of admission after 10 min of rest in a sitting position. ICG is a non-invasive technology that measures the total electrical conductivity of the thorax and its changes over time. It detects impedance changes caused by a high-frequency, low-magnitude current flowing through the thorax between two additional pairs of electrodes located outside of the measured segment. ICG provides information about heart rate (HR), blood pressure (BP), systemic vascular resistance index (SVRI), cardiac index (CI), and thoracic fluid content (TFC). TFC is an especially important parameter, as it indicates the extent of fluid accumulation in the chest, which could be useful in ADHF treatment as a marker of congestion [14,16]. The advantage of this method is that it is inexpensive and can be performed at the bedside. ICG is useful in differentiating the causes of dyspnea in emergency settings [17] and in predicting HF decompensation [16]. Echocardiography was conducted using Vivid S6 (GE-Healthcare, Chicago, IL, USA) and Vivid 7 (GE-Healthcare, Chicago, IL, USA) ultrasound systems. The standard assessment included the left ventricular ejection fraction (LVEF). There was no defined time limit for echocardiography, but the median time delay from admission to echocardiography was 3 days. Laboratory tests from peripheral venous blood samples were collected twice within 2 h of admission. The analysis included levels of N-terminal pro-brain natriuretic peptide (NTproBNP), hemoglobin, creatinine, and estimated glomerular filtration rate (eGFR), as calculated using the Cockcroft–Gault equation [18].

This analysis included 97 patients whose entire courses of intravenous diuretic dosing were precisely documented. For the purpose of this analysis, the patients were stratified into two subgroups based on the median value of intravenous (i.v.) diuretic utilization (equivalent to 540 mg furosemide i.v.): high-loop diuretic utilization (LDU, *n* = 49) and low-LDU (*n* = 48). Loop diuretic i.v. doses were assessed during the course of the entire hospitalization, and furosemide dose equivalents were calculated, with 1 mg of torsemide considered equivalent to 4 mg of furosemide [19].

The statistical analysis was performed using Statistica 12.0 (StatSoft, Inc., Tulsa, OK, USA). The distribution and normality of the data were assessed via visual inspection and the Kolmogorov–Smirnov test. Continuous variables were presented as the mean ± standard deviation (SD). Categorical variables were presented as absolute and relative frequencies (percentages). For comparative analysis, the study group was stratified into high-LDU and low-LDU groups. These subgroups were compared in terms of clinical, laboratory, and hemodynamic parameters using Student’s *t*-test or Mann–Whitney U-test for continuous variables and chi-squared or Fisher’s exact test for categorical variables. Logistic regression analysis was performed for the variables identified as differentiating the subgroups. A *p*-value of <0.05 was considered statistically significant.

## 3. Results

### 3.1. Study Group Baseline Characteristics

The study population consisted of 102 patients, of whom 78 (76.5%) were male, with a mean LVEF of 37.3 ± 14.1%. The majority of patients presented with a symptom severity of NYHA class III (*n* = 66, 64.7%), whereas the remaining 36 patients (35.3%) were of NYHA class IV [20]. The most commonly reported symptoms were dyspnea on exertion, orthopnea, and edema (Table 1). Ischemic heart disease, hypertension, atrial fibrillation, and valvular heart disease were the most common concomitant diseases (Table 1). Among the study population, 27 patients (26.5%) presented with de novo ADHF.

Most patients were treated according to the guidelines [1] with angiotensin-converting enzyme inhibitors (ACEIs) or angiotensin receptor blockers (ARBs) (70.6%), mineralocorticoid receptor antagonists (MRAs) (32.4%), beta-blockers (76.5%), and diuretics (72.5%). The demographic, baseline, and laboratory tests at admission are summarized in Table 1.

### 3.2. Comparison of Admission Characteristics of Patients Based on Diuretic Usage

**Figure 1:**
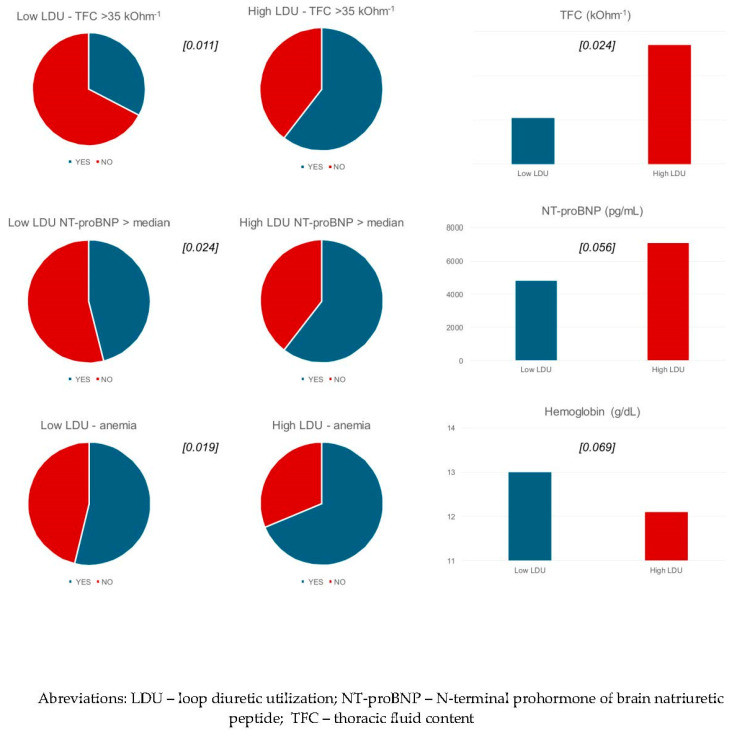
The most distinctive differences between low- and highLDU groups (*p* value in brackets).

The full course of i.v. diuretic dosing during hospitalization was documented for 97 patients, 49 of whom required high-LDU and 48 who required low-LDU treatment. Compared to low-LDU patients, high-LDU patients had higher TFC at admission, both quantitatively (37.4 ± 8.1 vs. 34.1 ± 6.9 kOhm-1; *p* = 0.024) and qualitatively (TFC ≥ 35 kOhm-1: 59.2% vs. 33.3%; *p* = 0.011). Furthermore, in the high-LDU group, anemia was more common (67.4% vs. 43.8%; *p* = 0.019), as was qualitatively elevated NT-proBNP (≥median of 3952 pg/mL: 60.4% vs. 37.5%; *p* = 0.024) (Table 2, Figure 1). No significant differences were observed in age; gender; NYHA class; symptoms; comorbidities (including diabetes, hypertension, and atrial fibrillation); pre-admission treatment, including diuretics, LVEF, HR, CI, BP, and SVRI; or chest X-ray changes. The high-LDU group was associated with significantly longer hospital stays (12.9 ± 6.4 vs. 7.0 ± 2.6 days; *p* < 0.001) (Table 2). 

Logistic regression analysis identified anemia, elevated NT-proBNP, and high thoracic fluid content as predictors of high LDU (HR: 2.65, 2.54, and 2.90, respectively). In a multifactorial model, only high TFC remained an independent predictor (HR: 2.60, 95% CI 1.04–6.49; *p* = 0.038) (Table 3).

## 4. Discussion

In this study, we demonstrated that TFC was the only independent admission marker of a high requirement for i.v. diuretics in patients hospitalized with decompensated heart failure.

Achieving decongestion is challenging because of the difficulties in assessing the diuretic response and complications from renal dysfunction, hypotension, and other general conditions [21]. It has been reported that 30% of patients with ADHF present with DR [22]. Importantly, the definition of DR has changed over time [23], highlighting the clinical challenges of this issue. DR can be defined as impaired sensitivity to diuretics, resulting in reduced natriuresis and diuresis, which limits the possibility of achieving euvolemia [7,24]. The simplest way to assess the response to diuretics is by measuring changes in body weight and fluid output, but doing so only indicates DR after several days of follow-up and is not free from limitations. It is recognized that the female sex, hypokalemia, hyponatremia, pulmonary infection, type 2 diabetes mellitus, kidney disease [13], high concentrations of NT-proBNP [13,25], and intra-abdominal hypertension [26] are risk factors associated with DR development.

In this secondary analysis, we assumed high LDU to be a proxy for DR. The intensity of congestion assessed by ICG was revealed to be the most distinctive feature of DR. However, high-LDU patients also had higher concentrations of creatinine, NT-proBNP, and a higher frequency of anemia. In contrast, we did not observe differences between the groups in terms of systolic and diastolic blood pressure, as had been observed in others’ research, despite comparable levels of NT-proBNP [13,25]. In accordance with a previous study, anemia was significantly more frequent in the high-LDU group [13], which was surprisingly not observed in the other papers [25]. Multiple studies have also highlighted that diabetics need to be treated with loop diuretics more intensively, both acutely and chronically [27,28]. Cunha et al. demonstrated that diabetic patients had 24% higher odds of requiring high-dose furosemide upon admission and independently 26% higher odds of being discharged with a prescription for at least 80 mg of furosemide per day [28]. In our analysis, we did not identify a relationship between diabetes and diuretic requirements. Our findings are also inconsistent with prior observations that NYHA class is associated with DR [13,29]. It is noteworthy that in our cohort, the severity of fluid overload, as assessed by the NYHA classification or observed on the chest X-ray, is not correlated with diuretic requirements. This suggests that TFC may serve as a more objective marker of congestion and indicates the need for a more aggressive diuretic treatment.

Moreover, the role of hyponatremia has been previously suggested as related to a higher degree of congestion and a higher requirement for loop diuretics during hospitalization [30]. This variable was not included in our analysis due to the low incidence of hyponatremia in the study group.

Many studies focus on the search for the earliest and best factor associated with DR. In the paper by Damman et al., they showed that natriuresis-guided diuretic treatment improved diuresis and natriuresis, irrespective of baseline eGFR and occurrence of worsening renal function (WRF); what is more, it was effective even in patients with low eGFR [29]. On the other hand, the study, whose aim was to identify the most accurate marker for early prediction of poor diuretic response in acute heart failure, demonstrated that urine Na+ adjusted for urine creatinine (UNa+/UCr ratio) and outperformed other markers, including spot urine sodium [31]. Since the recruitment to our study, the recommendations for the treatment of HF have been modified; in our group, patients were not treated with SGLT2 inhibitors, which we now know through their glucose and natriuretic effects have a beneficial effect on breaking DR [32].

The relationship between TFC and the need for diuretics is consistent with the experience of the use of remote assessment of pulmonary artery pressure, also a marker of pulmonary congestion. The monitoring of pulmonary artery pressure by CardioMEMS and, on this basis, the modification of diuretic and treatment had a beneficial effect on reducing the risk of hospitalization in patients with CHF [33]. It is worth mentioning that the remote monitoring of weight, as an indicator of volemia, was less effective regarding the risk of hospitalizations [34].

Our findings highlight the importance of personalizing diuretic treatment based on objective and complex clinical assessments. Patients with a higher TFC at admission should be suspected of having a higher diuretic requirement and will probably need to be more intensively treated. Our findings should be interpreted in the context of the current guidelines that recommend natriuresis-guided decongestion therapy [35].

## 5. Limitations

The findings of our study should be interpreted in light of several limitations. Firstly, it is a secondary analysis based on a single-center study with a limited participant pool, which may have affected the statistical power of certain comparisons. A small sample size can lead to potential biases and limit the generalizability of the findings, reducing the robustness of the conclusions drawn from our data. Moreover, the fact that our study was conducted at a single center meant that the results may not be applicable to broader populations or different clinical settings, necessitating caution in extrapolating our findings.

The exclusion of patients with anemia was especially important due to the previously reported association of anemia with fluid overload in ADCHF patients [36]. The exclusion of patients with mild and more advanced anemia limited this bias.

Additionally, natriuresis was not routinely measured in the clinical practice at our site during the study, potentially omitting a critical parameter that could provide further insight into the diuretic efficacy and fluid management strategies employed. This lack of routine measurement may obscure the relationship between diuretic therapy and patient outcomes, as natriuretic responses can be important indicators of renal function and fluid balance.

It is also noteworthy that ICG assessments were performed within a 24 h window, which may be significant given that hemodynamic profiles can fluctuate rapidly, sometimes within an hour of initiating diuretic treatment. This temporal limitation could affect the accuracy of our hemodynamic evaluations and their correlation with fluid management strategies.

Furthermore, comparisons with other studies should take into account that our analysis assessed the total hospital dose of diuretics administered, whereas most other research focused solely on diuretic dosing during the initial treatment period of 6 to 48 h. This distinction is critical, as total dosing may reflect different clinical practices and treatment paradigms that could influence patient outcomes.

It is also important to acknowledge that TFC is impacted by the presence of fluid in the pericardial or pleural cavities, indicating that it does not merely reflect pulmonary congestion. Likewise, the precision of ICG measurements may be compromised in cases of aortic regurgitation or atrial fibrillation with rapid ventricular rates, further complicating the interpretation of the hemodynamic data [37,38]. These factors underscore the necessity for future multicenter studies with larger cohorts to validate our findings and explore the complexities of diuretic management in various clinical scenarios.

## 6. Conclusions

TFC was the sole independent admission marker of high requirements for i.v. diuretics in patients hospitalized due to decompensated heart failure. An objective assessment of volume status by impedance cardiography may support intensive personalized decongestion therapy. Baseline high TFC may substantiate more aggressive decongestion therapy, potentially influencing the length of hospitalization. A further prospective and randomized study would be necessary to verify this hypothesis.

## Figures and Tables

**Table 1:** Baseline characteristics of the study group (*n* = 102).

Age (years)	71.4 ± 12.5
Male	78 (76.5%)
HR (bpm)	87 ± 24
Systolic BP (mmHg)	135 ± 27
Diastolic BP (mmHg)	82 ± 14
De novo HF	27 (26.5%)
**Symptoms and signs, *n* (%)**	
Dyspnea at rest	41 (40.2%)
Dyspnea on effort	100 (98.1%)
Orthopnea	78 (77.2%)
Paroxysmal nocturnal dyspnea	44 (43.1%)
Chest pain	25 (24.5%)
Palpitations	33 (32.4%)
Edema	77 (75.5%)
Tachypnea	21 (20.6%)
Ascites	16 (15.7%)
Peripheral hypoperfusion	10 (9.8%)
Hepatomegaly	18 (17.6%)
**Concomitant disease, *n* (%)**	
Prior myocardial infarction	42 (41.1%)
Hypertension	68 (66.6%)
Atrial fibrillation	54 (52.9%)
Type 2 diabetes mellitus	50 (49.0%)
Chronic kidney disease (stage ≥ 3)	30 (29.4%)
**Laboratory data on admission, mean ± SD**	
NT-proBNP (pg/mL)	6197 ± 7057
Creatinine (mg/dL)	1.31 ± 0.51
eGFR (mL/min/1.73 m^2^)	62.2 ± 23.9
Hemoglobin (g/dL)	12.6 ± 2.6

eGFR—estimated glomerular filtration rate, HF—heart failure, HR—heart rate, NT-proBNP—N-terminal pro-brain natriuretic peptide.

**Table 2:** Comparison of subgroups stratified by level of utilization of intravenous diuretics (*n* = 97).

	Low LDU (*n* = 48)	High LDU (*n* = 49)	*p*
**Demographic characteristic, mean ± SD or *n* (%)**			
Age (years)	70.8 ± 14.0	71.5 ± 11.1	0.917
Male	37 (77.1%)	37 (75.5%)	0.855
BMI (kg/m^2^)	29.2 ± 4.9	31.3 ± 7.7	0.105
NYHA Class III/IV	35 (72.9)/13 (27.1)	28 (57.1%)/21 (42.9%)	0.104
dnHF	14 (29.2%)	11 (22.5%)	0.449
**Medical history, *n* (%)**			
Prior MI	16 (33.3%)	23 (46.9%)	0.171
Hypertension	35 (72.9%)	30 (61.2%)	0.221
AF	23 (47.9%)	31 (63.3%)	0.128
Diabetes mellitus	21 (43.8%)	27 (55.1%)	0.263
COPD	8 (16.7%)	7 (14.3%)	0.745
CKD	10 (21.3%)	19 (38.9%)	0.062
Dyspnea at rest	17 (35.4%)	22 (44.9%)	0.341
Dyspnea during exercise	48 (100%)	48 (98%)	0.320
Orthopnea	36 (76.6%)	40 (81.6%)	0.543
Palpitations	16 (33.3%)	15 (30.6%)	0.774
Edema	37 (77.1%)	41 (83.7%)	0.414
**Clinical signs reported during examination, *n* (%)**			
Edema	38 (79.1%)	37 (75.5%)	0.667
Ascites	7 (14.6%)	9 (18.4%)	0.616
Tachypnea	6 (12.5%)	13 (26.5%)	0.082
Peripheral hypoperfusion	4 (8.3%)	6 (12.2%)	0.526
Hepatomegaly	8 (16.6%)	9 (18.4%)	0.826
**Treatment before admission, *n* (%)**			
Diuretics	35 (74.5%)	38 (79.2%)	0.587
ACE-I	32 (68.1%)	28 (58.3%)	0.235
ARB	5 (10.6%)	5 (10.4%)	0.972
BB	38 (80.9%)	36 (75%)	0.492
MRA	15 (31.9%)	18 (37.5%)	0.568
**Laboratory tests at admission, mean ± SD or *n* (%)**			
Creatinine (mg/dL)	1.194 ± 0.3	1.427 ± 0.6	0.028
eGFR (mL/min/1.73 m^2^)	74.6 ± 34.5	68.4 ± 37.0	0.362
NT-proBNP (pg/mL)	4820 ± 6025	7084 ± 7050	0.056
NT-proBNP (>median 3952 pg/mL)	18 (37.5%)	29 (60.4%)	0.024
Hemoglobin (g/dL)	13.0 ± 1.9	12.1 ± 2.5	0.069
Anemia (Hemoglobin < 13 g/dL for males or <12 g/dL for females)	21 (43.8%)	33 (67.4%)	0.019
**Hemodynamics, *n* (%) or mean ± SD**			
Congestion in chest X-ray	34 (73.9%)	39 (81.3%)	0.393
LVEF (%)	40.0 ± 14.6	35.6 ± 12.96	0.150
Reduced LVEF (<40%)	25 (53.2%)	31 (68.9%)	0.123
HR (bpm)	82 ± 22	81 ± 21	0.928
SBP (mmHg)	125 ± 27	121 ± 22	0.783
DBP (mmHg)	73 ± 13	73 ± 10	0.999
SI (mL/m^−2^)	40.3 ± 11.5	39.7 ± 15.0	0.435
CI (mL/m^2^/min)	3.1 ± 0.8	2.99 ± 0.9	0.333
SVRI (dyn/s/cm^5^/m^2^)	2283 ± 810	2363 ± 685	0.627
TFC (1/kOhm)	34.1 ± 6.9	37.4 ± 8.1	0.024
TFC > 35 (1/kOhm)	16 (33.3%)	29 (60.4%)	0.011
**Other variables, mean ± SD**			
Length of hospital stay (days)	6.97 ± 2.6	12.9 ± 6.3	<0.0001
i.v. loop diuretic dose (mg, furosemide equivalent)	299.8 ± 116.5	1486.9 ± 1371.0	<0.0001
Oral loop diuretic dose (mg, furosemide equivalent)	329 ± 322	479 ± 567	0.401

ACE-I—angiotensin-converting enzyme inhibitor; AF—atrial fibrillation; ARB—angiotensin receptor blocker; BB—beta-blocker, BMI—body mass index; CI—cardiac index; CKD—chronic kidney disease; COPD—chronic obstructive pulmonary disease; DBP—diastolic blood pressure; dnHF—de novo HF; eGFR—estimated glomerular filtration rate (by Cockcroft–Gault formula); HR—heart rate; i.v.—intravenous; LDU—loop diuretic utilization; LVEF—left ventricle ejection fraction; MI—myocardial infarction; MRA—mineralocorticoid receptor antagonist; NT-proBNP—N-terminal prohormone of brain natriuretic peptide; NYHA—New York Heart Association; SBP—systolic blood pressure; SI—stroke index; SVRI—systemic vascular resistance index; TFC—thoracic fluid content.

**Table 3:** Logistic regression results.

	Univariate Regression			Multivariate Regression		
	HR	95% CI	*p*	HR	95% CI	*p*
CKD	2.34	0.94–5.86	0.065	-	-	-
tachypnoe	2.53	0.86–7.43	0.088	-	-	-
anemia	2.65	1.15–6.12	0.021	-	-	-
NT-proBNP *	2.54	1.11–5.85	0.026	-	-	-
TFC > 35 1/kOhm	2.90	1.25–6.70	0.012	2.60	1.04–6.49	0.038

* over median. CKD—chronic kidney disease, NT-proBNP—N-terminal pro-brain natriuretic peptide, TFC—thoracic fluid content.

## Data Availability

The original contributions presented in the study are included in the article, further inquiries can be directed to the corresponding author.

## References

[1] McDonagh T.A., Metra M., Adamo M., Gardner R.S., Baumbach A., Böhm M., Burri H., Butler J., Čelutkienė J., Chioncel O. (2023). 2023 Focused Update of the 2021 ESC Guidelines for the diagnosis and treatment of acute and chronic heart failure. Eur. Heart J..

[2] McDonagh T.A., Metra M., Adamo M., Gardner R.S., Baumbach A., Böhm M., Burri H., Butler J., Čelutkienė J., Chioncel O. (2021). 2021 ESC Guidelines for the diagnosis and treatment of acute and chronic heart failure. Eur. Heart J..

[3] Heidenreich P.A., Bozkurt B., Aguilar D., Allen L.A., Byun J.J., Colvin M.M., Deswal A., Drazner M.H., Dunlay S.M., Evers L.R. (2022). 2022 AHA/ACC/HFSA Guideline for the Management of Heart Failure: A Report of the American College of Cardiology/American Heart Association Joint Committee on Clinical Practice Guidelines. J. Am. Coll. Cardiol..

[4] Hollenberg S.M., Warner Stevenson L., Ahmad T., Amin V.J., Bozkurt B., Butler J., Davis L.L., Drazner M.H., Kirkpatrick J.N., Peterson P.N. (2019). 2019 ACC Expert Consensus Decision Pathway on Risk Assessment, Management, and Clinical Trajectory of Patients Hospitalized with Heart Failure: A Report of the American College of Cardiology Solution Set Oversight Committee. J. Am. Coll. Cardiol..

[5] Urbich M., Globe G., Pantiri K., Heisen M., Bennison C., Wirtz H.S., Di Tanna G.L. (2020). A Systematic Review of Medical Costs Associated with Heart Failure in the USA (2014–2020). Pharmacoeconomics.

[6] Savarese G., Becher P.M., Lund L.H., Seferovic P., Rosano G.M.C., Coats A.J.S. (2023). Global burden of heart failure: A comprehensive and updated review of epidemiology. Cardiovasc. Res..

[7] Mullens W., Damman K., Harjola V.P., Mebazaa A., Brunner-La Rocca H.P., Martens P., Testani J.M., Tang W.H.W., Orso F., Rossignol P. (2019). The use of diuretics in heart failure with congestion—A position statement from the Heart Failure Association of the European Society of Cardiology. Eur. J. Heart Fail..

[8] Felker G.M., Ellison D.H., Mullens W., Cox Z.L., Testani J.M. (2020). Diuretic Therapy for Patients with Heart Failure: JACC State-of-the-Art Review. J. Am. Coll. Cardiol..

[9] Matsue Y., Damman K., Voors A.A., Kagiyama N., Yamaguchi T., Kuroda S., Okumura T., Kida K., Mizuno A., Oishi S. (2017). Time-to-Furosemide Treatment and Mortality in Patients Hospitalized with Acute Heart Failure. J. Am. Coll. Cardiol..

[10] Hodson D.Z., Griffin M., Mahoney D., Raghavendra P., Ahmad T., Turner J., Wilson F.P., Tang W.H.W., Rao V.S., Collins S.P. (2019). Natriuretic Response Is Highly Variable and Associated with 6-Month Survival: Insights from the ROSE-AHF Trial. JACC Heart Fail..

[11] Cox Z.L., Testani J.M. (2020). Loop diuretic resistance complicating acute heart failure. Heart Fail. Rev..

[12] Greene S.J., Triana T.S., Ionescu-Ittu R., Burne R.M., Guérin A., Borentain M., Kessler P.D., Tugcu A., DeSouza M.M., Felker G.M. (2020). In-Hospital Therapy for Heart Failure with Reduced Ejection Fraction in the United States. JACC Heart Fail..

[13] Lu X., Xin Y., Zhu J., Dong W., Guan T.P., Li J.Y., Li Q. (2022). Diuretic Resistance Prediction and Risk Factor Analysis of Patients with Heart Failure During Hospitalization. Glob. Heart..

[14] Galas A., Krzesiński P., Gielerak G., Piechota W., Uziębło-Życzkowska B., Stańczyk A., Piotrowicz K., Banak M. (2019). Complex assessment of patients with decompensated heart failure: The clinical value of impedance cardiography and N-terminal pro-brain natriuretic peptide. Heart Lung.

[15] Galas A., Krzesiński P., Banak M., Gielerak G. (2023). Hemodynamic Differences between Patients Hospitalized with Acutely Decompensated Chronic Heart Failure and De Novo Heart Failure. J. Clin. Med..

[16] Krzesiński P., Jankowska E.A., Siebert J., Galas A., Piotrowicz K., Stańczyk A., Siwołowski P., Gutknecht P., Chrom P., Murawski P. (2022). Effects of an outpatient intervention comprising nurse-led non-invasive assessments, telemedicine support and remote cardiologists’ decisions in patients with heart failure (AMULET study): A randomised controlled trial. Eur. J. Heart Fail..

[17] Peacock W.F.I.V., Albert N.M., Kies P., White R.D., Emerman C.L. (2000). Bioimpedance monitoring: Better than chest X-ray for predicting abnormal pulmonary fluid?. Congest. Heart Fail..

[18] Szummer K., Evans M., Carrero J.J., Alehagen U., Dahlström U., Benson L., Lund L.H. (2017). Comparison of the Chronic Kidney Disease Epidemiology Collaboration, the Modification of Diet in Renal Disease study and the Cockcroft-Gault equation in patients with heart failure. Open Heart..

[19] Ballester M.R., Roig E., Gich I., Puntes M., Delgadillo J., Santos B., Antonijoan R.M. (2015). Randomized, open-label, blinded-endpoint, crossover, single-dose study to compare the pharmacodynamics of torasemide-PR 10 mg, torasemide-IR 10 mg, and furosemide-IR 40 mg, in patients with chronic heart failure. Drug Des. Devel Ther..

[20] Galas A., Krzesinski P., Gielerak G. (2024). Thoracic fluid content as a marker of high intravenous diuretic requirements in patients hospitalized for heart failure decompensation. Heart Failure & World Congress on Acute Heart Failure 2024, Proceedings of the Heart Failure Association of the ESC, Lizbon, Portugal, 11–14 May 2024.

[21] Cox Z.L., Siddiqi H.K., Stevenson L.W., Bales B., Han J.H., Hart K., Imhoff B., Ivey-Miranda J.B., Jenkins C.A., Lindenfeld J. (2023). Randomized controlled trial of urinE chemiStry guided aCute heArt faiLure treATmEnt (ESCALATE): Rationale and design. Am. Heart J..

[22] Wilcox C.S., Testani J.M., Pitt B. (2020). Pathophysiology of Diuretic Resistance and Its Implications for the Management of Chronic Heart Failure. Hypertension.

[23] Shah N., Madanieh R., Alkan M., Dogar M.U., Kosmas C.E., Vittorio T.J. (2017). A perspective on diuretic resistance in chronic congestive heart failure. Ther. Adv. Cardiovasc. Dis..

[24] Gupta R., Testani J., Collins S. (2019). Diuretic Resistance in Heart Failure. Curr. Heart Fail. Rep..

[25] Imiela T., Imiela A.M., Karczmarewicz G., Budaj A. (2021). Acidic urine as a novel risk factor for diuretic resistance and worse in-hospital prognosis in patients with acute heart failure. Pol. Arch. Intern. Med..

[26] Nguyen V.Q., Gadiraju T.V., Patel H., Park M., Le Jemtel T.H., Jaiswal A. (2016). Intra-abdominal Hypertension: An Important Consideration for Diuretic Resistance in Acute Decompensated Heart Failure. Clin. Cardiol..

[27] Cubbon R.M., Adams B., Rajwani A., Mercer B.N., Patel P.A., Gherardi G., Gale C.P., Batin P.D., Ajjan R., Kearney L. (2013). Diabetes mellitus is associated with adverse prognosis in chronic heart failure of ischaemic and non-ischaemic aetiology. Diab. Vasc. Dis. Res..

[28] Cunha F.M., Pereira J., Marques P., Ribeiro A., Bettencourt P., Lourenço P. (2020). Diabetic patients need higher furosemide doses: A report on acute and chronic heart failure patients. J. Cardiovasc. Med..

[29] Damman K., Beldhuis I.E., van der Meer P., Krikken J.A., Coster J.E., Nieuwland W., van Veldhuisen D.J., Voors A.A., Ter Maaten J.M. (2024). Renal function and natriuresis-guided diuretic therapy—A pre-specified analysis from the PUSH-AHF trial. Eur. J. Heart Fail..

[30] Omar H.R., Guglin M. (2020). Higher Diuretic Requirements in Acute Heart Failure with Admission Hyponatraemia Versus Normonatraemia. Heart Lung Circ..

[31] Iwanek G., Guzik M., Zymliński R., Fudim M., Ponikowski P., Biegus J. (2024). Spot urine sodium-to-creatinine ratio surpasses sodium in identifying poor diuretic response in acute heart failure. ESC Heart Fail..

[32] Stachteas P., Nasoufidou A., Patoulias D., Karakasis P., Karagiannidis E., Mourtzos M.A., Samaras A., Apostolidou X., Fragakis N. (2024). The Role of Sodium-Glucose Co-Transporter-2 Inhibitors on Diuretic Resistance in Heart Failure. Int. J. Mol. Sci..

[33] Abraham W.T., Adamson P.B., Bourge R.C., Aaron M.F., Costanzo M.R., Stevenson L.W., Strickland W., Neelagaru S., Raval N., Krueger S. (2011). CHAMPION Trial Study Group. Wireless pulmonary artery haemodynamic monitoring in chronic heart failure: A randomised controlled trial. Lancet.

[34] Ong M.K., Romano P.S., Edgington S., Aronow H.U., Auerbach A.D., Black J.T., De Marco T., Escarce J.J., Evangelista L.S., Hanna B. (2016). Effectiveness of Remote Patient Monitoring after Discharge of Hospitalized Patients with Heart Failure: The Better Effectiveness after Transition—Heart Failure (BEAT-HF) Randomized Clinical Trial. JAMA Intern. Med..

[35] Dauw J., Charaya K., Lelonek M., Zegri-Reiriz I., Nasr S., Paredes-Paucar C.P., Borbély A., Erdal F., Benkouar R., Cobo-Marcos M. (2024). Protocolized Natriuresis-Guided Decongestion Improves Diuretic Response: The Multicenter ENACT-HF Study. Circ. Heart Fail..

[36] Krzesiński P., Galas A., Gielerak G., Uziębło-Życzkowska B. (2020). Haemodynamic Effects of Anaemia in Patients with Acute Decompensated Heart Failure. Cardiol. Res. Pract..

[37] Bayram M., Yancy C.W. (2009). Transthoracic impedance cardiography: A noninvasive method of hemodynamic assessment. Heart Fail. Clin..

[38] Siebiert J. (2007). Impedance Cardiography Guide for Physicians.

